# Nitro­sonium nitrate (NO^+^NO_3_^−^) structure solution using *in situ* single-crystal X-ray diffraction in a diamond anvil cell

**DOI:** 10.1107/S2052252521000075

**Published:** 2021-02-05

**Authors:** Dominique Laniel, Bjoern Winkler, Egor Koemets, Timofey Fedotenko, Stella Chariton, Victor Milman, Konstantin Glazyrin, Vitali Prakapenka, Leonid Dubrovinsky, Natalia Dubrovinskaia

**Affiliations:** a Material Physics and Technology at Extreme Conditions, Laboratory of Crystallography, University of Bayreuth, Bayreuth, 95440, Germany; b Institut für Geowissenschaften, Abteilung Kristallographie, Johann Wolfgang Goethe-Universität Frankfurt, Altenhöferallee 1, Frankfurt am Main, D-60438, Germany; c Bayerisches Geoinstitut, University of Bayreuth, Bayreuth, 95440, Germany; d Center for Advanced Radiation Sources, University of Chicago, Chicago, Illinois 60637, USA; e Dassault Systèmes BIOVIA, Cambridge, Cambridgeshire CB4 0WN, United Kingdom; f Photon Science, Deutsches Elektronen-Synchrotron, Notkestrasse 85, Hamburg, 22607, Germany; g Department of Physics, Chemistry and Biology (IFM), Linköping University, Linköping, SE-581 83, Sweden

**Keywords:** nitro­sonium nitrate, high-pressure single-crystal X-ray diffraction, positively charged oxygen atoms, structure refinement

## Abstract

At high pressures, autoionization – along with polymerization and metallization – is one of the responses of simple molecular systems to a rise in electron density. Nitro­sonium nitrate (NO^+^NO_3_^−^), known for this property, has attracted a large interest in recent decades and was reported to be synthesized at high pressure and high temperature from a variety of nitro­gen–oxygen precursors, such as N_2_O_4_, N_2_O and N_2_–O_2_ mixtures. However, its structure has not been determined unambiguously. Here, we present the first structure solution and refinement for nitro­sonium nitrate on the basis of single-crystal X-ray diffraction at 7.0 and 37.0 GPa. The structure model (*P*2_1_/*m* space group) contains the triple-bonded NO^+^ cation and the NO_3_^−^ *sp* ^2^-trigonal planar anion. Remarkably, crystal-chemical considerations and accompanying density-functional-theory calculations show that the oxygen atom of the NO^+^ unit is positively charged – a rare occurrence when in the presence of a less-electronegative element.

## Abstract 2

The crystal structure of nitro­sonium nitrate (NO^+^NO_3_^−^) is determined by synchrotron single-crystal X-ray diffraction at pressures of 7.0 and 37.0 GPa – resolving a long-standing controversy. Remarkably, the oxygen atom of the NO^+^ unit is determined to be positively charged, a rare occurrence when in the presence of a less-electronegative element.

## 1. Introduction   

Characterizing the behavior of simple molecular systems has always been in the focus of high-pressure research as this enables us to deepen our understanding of the pressure dependence of interatomic interactions and to benchmark theoretical models. The studies of simple molecules, such as nitro­gen, carbon monoxide, carbon dioxide, oxygen, hydrogen and acetyl­ene, show common trends as a function of density. At low pressures, the intermolecular interactions are dominated by very weak dispersion forces (London/van der Waals, Keesom) so that the molecules are often completely or partially rotationally disordered. At slightly higher pressures, stronger interactions – *e.g.* intermolecular electric quadrupole–quadrupole or magnetic interactions – begin to dominate and force the molecules into specific alignments. On further volume reduction, other mechanisms redistribute the continuously rising electron density. At this stage, most simple molecular systems are found to either polymerize [N_2_ (Laniel *et al.*, 2020*b* ▸; Eremets *et al.*, 2004 ▸), CO (Evans *et al.*, 2006 ▸), CO_2_ (Dziubek *et al.*, 2018 ▸; Datchi *et al.*, 2009 ▸), C_2_H_2_ (Trout & Badding, 2000 ▸)] or metallize [O_2_ (Weck *et al.*, 2002 ▸), H_2_ (Loubeyre *et al.*, 2020 ▸; Wigner & Huntington, 1935 ▸)]. In contrast, N_2_O_4_, N_2_O and N_2_–O_2_ mixtures are among the simple molecular systems which deviate from this typical pressure-induced behavior and instead favor another pathway, *i.e.* ionization. Indeed, all the aforementioned nitro­gen–oxygen systems are thought to transform into the ionic solid nitro­sonium nitrate (NO^+^NO_3_^−^) (Somayazulu *et al.*, 2001 ▸; Agnew *et al.*, 1983 ▸).

The first report of the synthesis of nitro­sonium nitrate, albeit in liquid form, dates back to 1948, when it was produced by the low-temperature heterolytic dissociation of a N_2_O_4_ precursor in a solvent with a high dielectric constant (Addison & Thompson, 1948 ▸). Its solid-state temperature-driven formation from NO was achieved in 1965 at low temperatures (Parts & Miller, 1965 ▸), while its pressure-driven synthesis was shown in 1983 through the compression of β-N_2_O_4_ to 0.3 GPa in a diamond anvil cell (DAC) (Agnew *et al.*, 1983 ▸). Its synthesis from N_2_O was showcased more recently, in 2001, at a pressure of 20 GPa and a temperature of 1000 K (Somayazulu *et al.*, 2001 ▸). From the many experimental investigations performed at high pressures (Somayazulu *et al.*, 2001 ▸; Agnew *et al.*, 1983 ▸, 1985 ▸; Meng *et al.*, 2006 ▸; Yoo *et al.*, 2003 ▸; Kuznetsov *et al.*, 2008 ▸; Song *et al.*, 2003*a* ▸,*b* ▸; Sihachakr & Loubeyre, 2006 ▸), nitro­sonium nitrate was deduced to be stable from 1.7 to at least 55 GPa. It is also thought to be the thermodynamically stable phase of the oxygen–nitro­gen binary under these pressure–temperature conditions. A change of slope in the pressure evolution of IR and Raman modes above 5 GPa at 80 K, as well as the disappearance of Raman modes, suggested a subtle phase transition (Song *et al.*, 2003*a* ▸,*b* ▸); tentatively assigned to a shift from rotationally disordered ions to ordered ions. Notably, across the many studies of NO^+^NO_3_^−^ – and independently of the precursors used – the same Raman modes were consistently and reproducibly detected.

Still, despite the many experimental investigations leading to the spectroscopic identification of nitro­sonium nitrate, no consensus has yet been reached on its crystal structure. Numerous powder X-ray diffraction (pXRD) studies have reported an orthorhombic structure and, based on extinction rules, suggested space groups *Pmcn* (Somayazulu *et al.*, 2001 ▸; Song *et al.*, 2003*b* ▸), *P*2_1_ *cn* (Somayazulu *et al.*, 2001 ▸; Song *et al.*, 2003*b* ▸), *Pnma* (Yoo *et al.*, 2003 ▸), *Pn*2_1_ *a* (Yoo *et al.*, 2003 ▸) or *Pmmm* (Sihachakr & Loubeyre, 2006 ▸). Such a diversity is caused by the difficulty of the analysis of the pXRD patterns, which usually feature not only the diffraction lines of NO^+^NO_3_^−^ but also those of its precursors (N_2_O, N_2_O_4_, N_2_–O_2_) or pure N_2_, which strongly overlap with those of nitro­sonium nitrate. This has also prevented an accurate determination of the compound’s unit-cell parameters. However, Meng *et al.* (2006 ▸) produced NO^+^NO_3_^−^ at 1.7 GPa from a mixture of N_2_ and O_2_ irradiated by hard X-rays and were able to collect higher-quality pXRD data. From it, a structural model with a monoclinic unit cell (*P*2_1_/*m* space group) and the atomic positions of the NO^+^ and NO_3_^−^ species was proposed. Later theoretical calculations cast a shadow on this structural model as it was found to be less stable than another model with the space group *P*2_1_ and a different orientation of the NO^+^ cations (Li *et al.*, 2015 ▸).

Here, we present the results of our synchrotron X-ray diffraction and Raman spectroscopy investigation of nitro­sonium nitrate, synthesized from laser-heated N_2_O, in the pressure range of 3.9 to 55.0 GPa. At the pressure points of 7.0 and 37.0 GPa, single-crystal X-ray diffraction datasets were collected and the structure of NO^+^NO_3_^−^ was solved and refined, thus resolving the long-standing dispute. These experimental data also allowed an accurate determination of the equation of state of nitro­sonium nitrate and demonstrated the striking presence of a positively charged oxygen atom in the NO^+^ ion. Complementing density-functional-theory (DFT) based calculations support the experimental findings.

## 2. Methods   

### 2.1. Experimental method   

To explore the NO^+^NO_3_^−^ system up to 55.0 GPa, DACs equipped with anvils with culet diameters of 250 µm were prepared. Nitrous oxide (N_2_O) was loaded cryogenically. Gold micrograins were put into the DAC and utilized as *in situ* pressure calibrants as well as YAG laser absorbers (Dewaele *et al.*, 2008 ▸). When the gold was irradiated using a low laser power, the sample produced no detectable thermoemission, but at a high power extremely intense flashes occurred. The very short time of the flashing prevented measuring the samples’ temperature but it was estimated to be higher than 2500 K. The gold was not observed to chemically react with the sample after laser heating.

Raman spectra were obtained by confocal Raman spectroscopy measurements performed with a LabRam spectrometer equipped with a ×50 Olympus long-working-distance objective. For the excitation, a continuous He–Ne laser (632.8 nm) with a focused laser spot of ∼2 µm in diameter was employed. The Stokes Raman signal was collected in a backscattering geometry using a CCD coupled to a 1800 lines mm^−1^ grating, allowing a spectral resolution of ∼2 cm^−1^.

The X-ray diffraction studies were performed at the GSECARS beamline (λ = 0.2952 Å) of the Advanced Photon Source (APS) as well as at the P02.2 Extreme Conditions beamline (λ = 0.2891 or 0.4808 Å) at PETRA III. Single-crystal X-ray diffraction (sc-XRDp) data were collected at various pressures between 7.0 and 55.0 GPa by rotating the DAC in step scans of 0.5° from −36 to +36° around the vertical axis. In sc-XRDp, ‘p’ designates micrometre- to submicrometre-sized single crystals that are usually characteristic of polycrystalline samples in conventional crystallography but here are instead used for diffraction studies of single crystals synthesized *in situ* in a DAC. The *CrysAlis PRO* software (Rigaku Oxford Diffraction, 2015 ▸) was utilized for the single-crystal data analysis (peak search, unit-cell finding, data integration), while the crystal structures were solved and refined with the *JANA2006* software (Petříček *et al.*, 2014 ▸). The sc-XRDp procedure for the data acquisition from micro-crystals and their analysis has previously been successfully employed (Laniel *et al.*, 2019*a* ▸,*b* ▸, 2020*a* ▸,*b* ▸) and the details can be found elsewhere (Bykova, 2015 ▸). Powder X-ray diffraction was also performed, and the pXRD data were integrated with *Dioptas* (Prescher & Prakapenka, 2015 ▸) and analyzed with the *XRDA* software (Desgreniers & Lagarec, 1994 ▸). A Le Bail unit-cell parameters refinement employing a pXRD pattern from NO^+^NO_3_^−^ at 3.9 GPa was accomplished with the *FullProf* software (Rodríguez-Carvajal, 1993 ▸). The pressure–volume data were fitted with a second-order Birch–Murnaghan equation of state using the *EoSFit7* software (Angel *et al.*, 2014 ▸).

### 2.2. Density-functional-theory-based calculations   

DFT calculations were performed using the *CASTEP* code (Clark *et al.*, 2005 ▸). The code is an implementation of Kohn–Sham DFT based on a plane-wave basis set in conjunction with pseudopotentials. The plane-wave basis set is unbiased (as it is not atom centered) and does not suffer from the problem of basis-set superposition error unlike atom-centered basis sets. It also makes converged results straightforward to obtain in practice, as the basis-set convergence is controlled by a single adjustable parameter, the plane wave cut-off. Pseudopotentials were either norm-conserving (then the cut-off was 1020 eV) or ultrasoft (the cut-off was 630 eV), and were generated using the Perdew–Burke–Ernzerhof (PBE) exchange-correlation functional (Perdew *et al.*, 1996 ▸) using the ‘on the fly’ parameters included in the *CASTEP* 2019 distribution. These pseudopotentials have been shown to be very accurate and are very well suited for the calculations carried out here (Lejaeghere *et al.*, 2016 ▸). The Brillouin-zone integrals were performed using Monkhorst–Pack grids (Monkhorst & Pack, 1976 ▸). with spacings between grid points of ∼0.025 Å^−1^. Full geometry optimizations of the unit-cell parameters and the internal coordinates were performed until forces were typically converged to <0.005 eV Å^−1^.

In our calculations, we neglect temperature, configurational entropy and the entropy contribution caused by lattice vibrations. Force and stress-free configurations obtained for a specific atomic configuration and a pre-set pressure correspond to local minima (metastable phases) or the global minimum (thermodynamically stable phase) of the enthalpy landscape. The use of DFT-based calculations for the prediction of structures and to cross-correlate structure–property relations is well established. Typically, DFT–GGA–PBE calculations (GGA = generalized gradient approximation), such as those carried out here, predict structural parameters to within 1–2%. Phonon-dispersion curves and Raman spectra are typically reproduced to within 5%.

## 3. Results   

**Figure 1:**
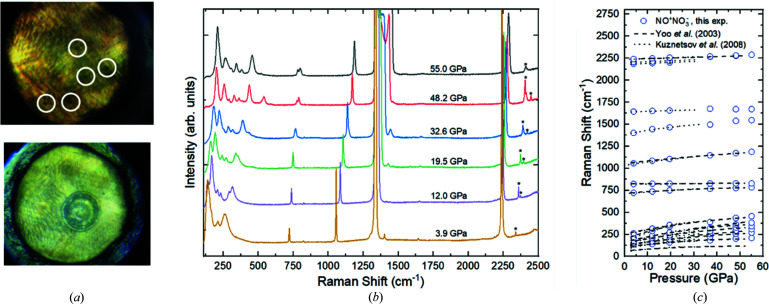
(*a*) Microphotographs of a N_2_O sample at 15.8 GPa, before (top) and after (bottom) laser heating. The gold particles are encircled in white (top). The recrystallized matter in the area surrounding the laser-heated gold particles is where the chemical reaction occurred (bottom). (*b*) Raman spectra of NO^+^NO_3_^−^ obtained upon the sample decompression from 55.0 down to 3.9 GPa. The Raman modes marked by asterisks belong to pure molecular nitro­gen (Bini *et al.*, 2000 ▸). (*c*) The evolution of the Raman shift of the nitro­sonium nitrate Raman modes compared with those previously reported (Yoo *et al.*, 2003 ▸; Kuznetsov *et al.*, 2008 ▸).

The N_2_O samples in the DACs were indirectly laser heated at pressures of 7.0, 15.8 and 37.0 GPa, as described above. As seen in Fig. 1 ▸(*a*), the material in the area surrounding the Au grains recrystallized, and a chemical reaction occurred, as confirmed by Raman spectroscopy. Indeed, the spectra obtained from this area show the Raman modes of the ionic nitro­sonium nitrate (NO^+^NO_3_^−^) and pure molecular nitro­gen, as expected from the decomposition of nitrous oxide N_2_O according to the reaction 4N_2_O → NO^+^NO_3_^−^ + 3N_2_. Figs. 1 ▸(*b*) and 1 ▸(*c*) show the evolution of the Raman spectra with pressure. The observed Raman modes of NO^+^NO_3_^−^ are in agreement with those previously reported for solid nitro­sonium nitrate (Yoo *et al.*, 2003 ▸; Kuznetsov *et al.*, 2008 ▸).

**Figure 2:**
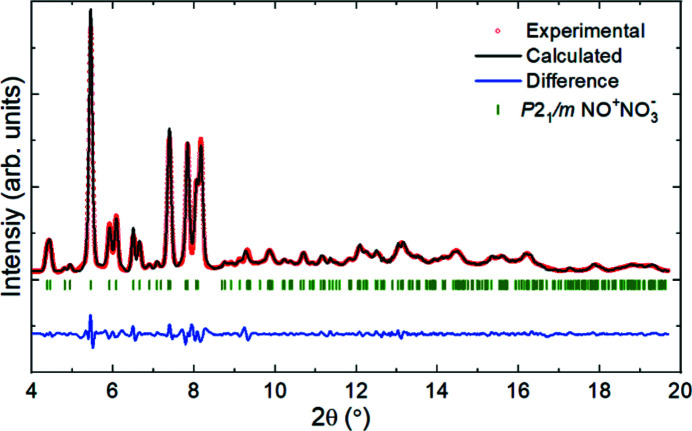
The diffraction pattern (λ = 0.2891 Å) of nitro­sonium nitrate at 3.9 GPa following the sample laser heating at 37.0 GPa and subsequent decompression. The red dots, black line and green ticks represent the experimental data, the Le Bail fit and the positions of the diffraction lines of nitro­sonium nitrate, respectively. The diffraction lines of β-N_2_ are not visible at this sample position.

**Figure 3:**
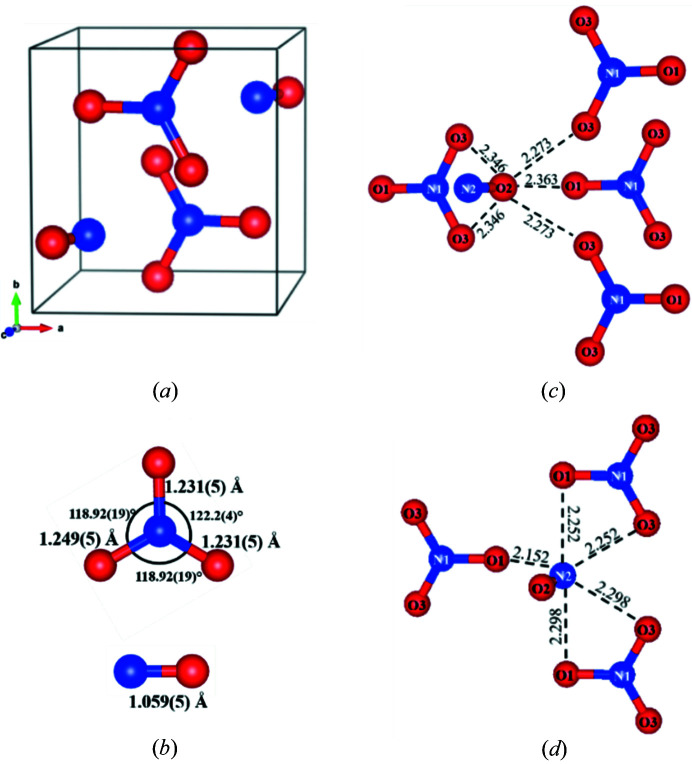
(*a*) A monoclinic unit cell of NO^+^NO_3_^−^ at 37 GPa. (*b*) A representation of the trigonal planar nitrate (top) and linear nitro­sonium (bottom) ions with bond lengths and bond angles indicated. (*c*)–(*d*) The environment of the O2 and N2 atoms forming the NO^+^ cation: both N2 and O2 atoms are fivefold coordinated by negatively charged oxygen atoms from the NO_3_^−^ anions. The lengths are provided in Å.

The transformed samples were also investigated by X-ray diffraction. The high-quality sc-XRDp data collected at 7.0 and 37.0 GPa allowed the crystal-structure solution and refinement. The orthorhombic unit cells, previously suggested by pXRD studies (Somayazulu *et al.*, 2001 ▸; Yoo *et al.*, 2003 ▸; Song *et al.*, 2003*b* ▸; Sihachakr & Loubeyre, 2006 ▸), were not confirmed. Instead, nitro­sonium nitrate was found to crystallize in a monoclinic structure (*P*12_1_/*m*1 space group, #11), with lattice parameters of *a* = 4.598 (3) Å, *b* = 4.975 (4) Å, *c* = 5.196 (8) Å and β = 96.39 (9)° [*V* = 118.1 (2) Å^3^] at 7.0 GPa, and *a* = 4.242 (2) Å, *b* = 4.595 (4) Å, *c* = 4.638 (2) Å and β = 94.70 (4)° [*V* = 90.08 (10) Å^3^] at 37.0 GPa. The position of all atoms at these two pressures was determined. As expected from the density increase, the refined atomic thermal parameters decrease, on average, between 7.0 and 37.0 GPa. The crystallographic data for NO^+^NO_3_^−^ are provided in Table 1 ▸. The Le Bail fit, performed on the pXRD data collected at 3.9 GPa (Fig. 2 ▸), shows that all observed diffraction lines belong to the nitro­sonium nitrate structure as determined by sc-XRDp. The structure solved with single-crystal data (see Fig. 3 ▸) validates the Raman measurements-based hypothesis that the compound is composed of two building blocks: nitro­sonium (NO^+^) and nitrate (NO_3_^−^). At 37 GPa, the single-crystal X-ray diffraction data were of sufficient quality to provide reliable interatomic distances. The nitro­sonium N–O bond length was found to be 1.059 (5) Å. This is identical, within uncertainty, to the 1.06 Å value typically measured at ambient conditions (Rosokha & Kochi, 2001 ▸; Andreev *et al.*, 2011 ▸; Tikhomirov *et al.*, 2002 ▸) and highlights that the triple bond of the oxygen–nitro­gen dimer is maintained even at such pressure. At the same pressure of 37 GPa, the nitrate anion has two N–O bond lengths of 1.231 (5) Å and one of 1.249 (5) Å, and two O–N–O angles of 118.92 (19)° and one of 122.2 (4)°. Its trigonal planar shape (the angles are almost equal to 120°) implies the *sp* ^2^-type hybridization of the nitro­gen atom. The refined angles and bond lengths are typical of NO_3_^−^ at ambient conditions (Tikhomirov *et al.*, 2002 ▸; Meyer *et al.*, 1976 ▸; Nimmo & Lucas, 1976 ▸).

As seen in Table 1 ▸, the agreement between the experimentally determined structure at 37 GPa and the DFT model is reasonable. The experimental and calculated N–O distances differ from each other within *ca*. 1–2%. The bond population for the nitro­sonium group is 1.03 eÅ^−3^, which confirms the presence of the strong covalent bonding that was inferred from the interatomic distances. Such good agreement shows that the DFT model describes the structure well and hence that this model can be employed to extract further information on the compound.

**Figure 4:**
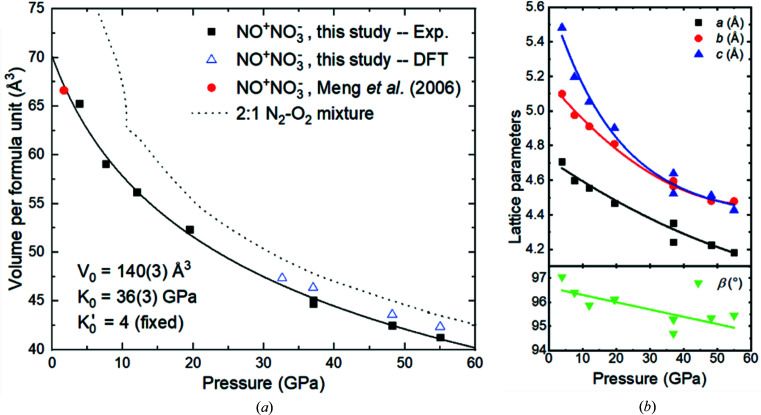
(*a*) Pressure dependence of the unit-cell volume per formula unit of nitro­sonium nitrate. The volume of a 2:1 N_2_–O_2_ mixture (Olijnyk, 1990 ▸; Akahama *et al.*, 1995 ▸; Gregoryanz *et al.*, 2007 ▸) is significantly larger than that of NO^+^NO_3_^−^ at corresponding pressures. The volume determined by Meng *et al.* (2006 ▸) (red dot) fits well the equation of state of nitro­sonium nitrate obtained in the current work. The data represented by the blue triangles were obtained by DFT calculations. (*b*) The lattice parameters of NO^+^NO_3_^−^ as a function of pressure.

Using both the sc-XRDp and pXRD data, the lattice parameters of NO^+^NO_3_^−^ were obtained in the pressure range of 3.9 to 55.0 GPa, which allowed an accurate determination of its equation of state (Fig. 4 ▸). By fitting the pressure–volume data with a second-order Birch–Murnaghan equation of state, the bulk modulus was found to be *K*_0_ = 36 (3) GPa. As expected for an ionic compound, this value is significantly higher than those known for most simple molecular compounds with dominating van der Waals intermolecular interactions (*i.e.* ɛ-N_2_, α-N_2_O, CO_2_-I, Xe(N_2_)_2_, with *K*_0_ < 10 GPa) (Yoo *et al.*, 2003 ▸; Laniel *et al.*, 2016 ▸; Olijnyk, 1990 ▸) but significantly lower in comparison with those of polymeric CO_2_ or cg-N (cubic gauche) (*K*_0_ > 110 GPa) (Eremets *et al.*, 2004 ▸; Dziubek *et al.*, 2018 ▸). Compared with a 2:1 N_2_–O_2_ mixture (Olijnyk, 1990 ▸; Akahama *et al.*, 1995 ▸; Gregoryanz *et al.*, 2007 ▸), the volume of nitro­sonium nitrate per formula unit is distinctly smaller. DFT-calculated volumes between 30 and 55 GPa match well with the experimental data.

The orientation of the NO^+^ species relative to the NO_3_^−^ species is shown in Figs. 3 ▸(*c*) and 3 ▸(*d*). In its first coordination shell, the nitro­gen atom (N2) of each nitro­sonium group has five oxygen atoms (three O1 and two O3) provided by three surrounding NO_3_^−^ anions. The intermolecular N2–O distances vary within *ca*. 2.15 to 2.30 Å. The oxygen atom (O2) of NO^+^ also has five oxygen atoms (one O1 and four O3) from the four closest NO_3_^−^ ions as its first intermolecular neighbors. The O2–O distances vary within *ca*. 2.27 to 2.36 Å and appear to be slightly greater than the N2–O distances owing to the larger size of the oxygen atom compared with that of the nitrogen atom. These observations highlight that both nitrogen and oxygen of the nitro­sonium units orient themselves towards negatively charged atoms, thus suggesting positive charges on both of them. Moreover, the O2–O3 distance of 2.27 Å is unusually short for oxygen (Ryan *et al.*, 2016 ▸; Liu *et al.*, 2007 ▸; Bergmann *et al.*, 2007 ▸) – even at such pressures or higher (Umemoto & Wentzcovitch, 2005 ▸; Bykova *et al.*, 2013 ▸, 2016 ▸) – which strengthens the claim of a positive charge on O2. A Mulliken population analysis shows that the nitro­gen in the nitro­sonium group has a smaller charge than that in the nitrate group (+0.42 versus +0.67 |e|). More interesting is the fact that the oxygen atoms in the nitro­sonium groups are indeed slightly positively charged (+0.05 |e|), while those in the nitrate group carry a charge of −0.38 |e|. A Hirshfeld analysis yields similar results, with a slightly higher positive charge on the oxygen of the nitro­sonium (+0.1 |e|). For oxygen, even a marginally positive effective charge is uncommon (Shudo *et al.*, 1981 ▸; Snyder & Fowler, 1993 ▸) – including that in NO^+^ cations (Andreev *et al.*, 2011 ▸) – although it is common in oxygen–fluorine compounds such as OF_2_ and O_2_F_2_ (Marx & Seppelt, 2015 ▸).

The structure of NO^+^NO_3_^−^ at 7.0 and 37.0 GPa, solved in the present work from sc-XRDp, is similar to the structure previously proposed by Meng *et al.* (2006 ▸) for nitro­sonium nitrate at 1.7 GPa (*P*2_1_/*m* space group). Indeed, when accounting for the change in the lattice parameters owing to the pressure difference, the sole distinction between the two is the exchanged positions of the oxygen and nitrogen atoms in the NO^+^ cation. In the Meng *et al.* (2006 ▸) model, if compared with ours, the oxygen atom occupies the position of the nitrogen atom and *vice versa*. However, if based on this model, the quality of our structure refinements decreases. Indeed, the *R*_1_ rises from 7.64 to 10.81% at 7.0 GPa and from 6.99 to 11.31% at 37.0 GPa. Moreover, switching the N2 atom to an oxygen atom – accordingly to the Meng *et al.* (2006 ▸) model – leads to an increase of its isotropic atomic displacement parameters (ADPs); suggesting a too large electron density on the site. The ADP goes from 0.026 (for N in the N2 position in our model) to 0.045 [for O in the same position in the Meng *et al.* (2006 ▸) model] at 7 GPa and from 0.017 to 0.027 at 37 GPa. Simultaneously, the O2 atom replaced by a nitro­gen atom has a decreased ADP. This provides an additional argument in favor of our model. Thus, the Meng *et al.* (2006 ▸) structural model can be ruled out for pressures between 7.0 and 37.0 GPa. Although this model still could be valid for the lower-pressure polymorph of NO^+^NO_3_^−^ (Meng *et al.*, 2006 ▸), crystal-chemical observations make this unlikely. A close inspection of the structure proposed at 1.7 GPa (Meng *et al.*, 2006 ▸) reveals the greater separation of the nitrogen atom of the nitro­sonium from its neighboring oxygen atoms than the separation of the oxygen atom from its oxygen neighbors. Given the larger size of the oxygen atom compared with the nitrogen atom, especially when accounting for the greater positive charge on the nitrogen atom, the configuration of the NO^+^ ion found in the present work is expected to be preferable. A structural phase transition from a low-pressure phase to a high-pressure phase of NO^+^NO_3_^−^ is thus deemed unlikely.

The DFT calculations also show that the Meng *et al.* (2006 ▸) model, at 1.7 GPa, is less stable than the one here proposed by ∼0.1 eV atom^−1^. However, the calculations also detected imaginary phonons at the Γ point for both structural models at pressures below 30 GPa, while above 30 GPa the structures are dynamically stable. This suggests that more complex calculations, for example accounting for anharmonicity, are required below that pressure to fully reproduce the experimental results. These are outside the scope of the present work. At 32.6 GPa, the previously proposed *P*2_1_ NO^+^NO_3_^−^ structure (Li *et al.*, 2015 ▸) – which has been calculated to be more stable than the Meng *et al.* (2006 ▸) model – is found to be ∼0.1 eV atom^−1^ higher in enthalpy (*i.e.* less stable) than the structural model proposed here.

## 4. Conclusions   

Nitro­sonium nitrate was here investigated using sc-XRDp, pXRD, Raman spectroscopy and DFT calculations in the pressure range of 3.9 to 55.0 GPa. High-quality single-crystal data were collected at 7.0 as well as at 37.0 GPa and enabled us to solve and refine the structure of NO^+^NO_3_^−^, which had been unknown for decades. Accompanying DFT calculations validated the stability of the experimentally determined structure, with a lower enthalpy than the previously derived models. The oxygen atom of the nitro­sonium cation was found to carry an unusual small positive charge, as suggested by crystal-chemical analysis and further verified by DFT calculations. These results highlight the significance of sc-XRDp as the most reliable method for the structural analysis at high pressures.

## Supplementary Material

Crystal structure: contains datablock(s) global, I. DOI: 10.1107/S2052252521000075/lt5036sup1.cif

CCDC reference: 2053666

## Figures and Tables

**Table:** Some parameters have both the experimental and the calculated value.

	NO^+^NO_3_^−^		NO^+^NO_3_^−^	
	Experimental	Calculated	Experimental	Calculated
Pressure (GPa)	7.0	7.0	37.0	37.0
Space group (#)	11	11	11	11
*Z*	2	2	2	2
*a* (Å)	4.598 (3)	4.5317	4.242 (2)	4.1869
*b* (Å)	4.975 (4)	4.9859	4.595 (4)	4.5970
*c* (Å)	5.196 (8)	5.5309	4.638 (2)	4.7988
β (°)	96.39 (9)	93.91	94.70 (4)	92.70
*V* (Å^3^)	118.1 (2)	124.680	90.08 (10)	92.259
Refinement details				
Wavelength (λ, Å)	0.2952		0.2891	
μ (mm^−1^)	0.064		0.084	
# Measured/independent reflections (*I* ≥ 3σ)	378/145 (109)		546/288 (154)	
(sin θ/λ)_max_ (Å^−1^)	0.858		1.036	
*R*_int_ (%)	5.12		3.80	
*R*_1_ (%)	7.64		6.99	
*wR*_2_ (%)	7.25		7.01	
*R*_1_ (all data, %)	9.19		9.65	
*wR*_2_ (all data, %)	7.34		7.85	
No. of parameters	17		17	
Δρ_min_, Δρ_max_ (eÅ^−3^)	−0.27, 0.38		−0.53, 0.50	

**Table:** 

7.0 GPa				
Atom	Wyckoff position	Fractional atomic coordinates (*x*, *y*, *z*)		*U*_iso_
N1	2*e*	0.5302 (11), 0.25, 0.260 (2)		0.0095 (12)
N2	2*e*	0.1659 (15), 0.25, 0.694 (3)		0.0263 (15)
O1	2*e*	0.8003 (10), 0.25, 0.305 (2)		0.0148 (10)
O2	2*e*	0.0569 (10), 0.25, 0.853 (2)		0.0212 (12)
O3	4*f*	0.3938 (7), 0.0329 (6), 0.2345 (14)		0.0179 (9)
				
37.0 GPa				
Atom	Wyckoff position	Fractional atomic coordinates (*x*, *y*, *z*)		*U*_iso_
		Experimental	Calculated	
N1	2*e*	0.5250 (8), 0.25, 0.2645 (7)	0.54420, 0.25, 0.27439	0.0151 (7)
N2	2*e*	0.1702 (8), 0.25, 0.6797 (7)	0.1858, 0.25, 0.69858	0.0166 (7)
O1	2*e*	0.8185 (6), 0.25, 0.3180 (6)	0.83256, 0.25, 0.34185	0.0138 (6)
O2	2*e*	0.0598 (6), 0.25, 0.8765 (5)	0.05893, 0.25, 0.89064	0.0156 (6)
O3	4*f*	0.3853 (4), 0.0155 (9), 0.2380 (4)	0.39896, 0.01802, 0.23878	0.0158 (5)
